# Mesenchymal stromal cell therapy reduces lung inflammation and vascular remodeling and improves hemodynamics in experimental pulmonary arterial hypertension

**DOI:** 10.1186/s13287-017-0669-0

**Published:** 2017-10-03

**Authors:** Lucas de Mendonça, Nathane S. Felix, Natália G. Blanco, Jaqueline S. Da Silva, Tatiana P. Ferreira, Soraia C. Abreu, Fernanda F. Cruz, Nazareth Rocha, Patrícia M. Silva, Vanessa Martins, Vera L. Capelozzi, Gizele Zapata-Sudo, Patricia R. M. Rocco, Pedro L. Silva

**Affiliations:** 1 Laboratory of Pulmonary Investigation, Carlos Chagas Filho Biophysics Institute, Federal University of Rio de Janeiro, Centro de Ciências da Saúde, Avenida Carlos Chagas Filho, s/n, Bloco G-014, Ilha do Fundão, Rio de Janeiro, RJ 21941-902 Brazil; 2 National Institute of Science and Technology for Regenerative Medicine, Rio de Janeiro, RJ Brazil; 3 Laboratory of Cardiovascular Pharmacology, Federal University of Rio de Janeiro, Rio de Janeiro, RJ Brazil; 4 Department of Physiology, Fluminense Federal University, Niterói, RJ Brazil; 5 Laboratory of Inflammation, Oswaldo Cruz Institute—Oswaldo Cruz Foundation, Rio de Janeiro, RJ Brazil; 6 Laboratory of Histomorphometry and Lung Genomics, University of São Paulo Faculty of Medicine, São Paulo, SP Brazil

**Keywords:** Pulmonary arterial hypertension, Mesenchymal stromal cells, Hemodynamics, Lung vascular remodeling, Macrophage phenotype

## Abstract

**Background:**

Experimental research has reported beneficial effects of mesenchymal stromal cell (MSC) therapy in pulmonary arterial hypertension (PAH). However, these studies either were based on prophylactic protocols or assessed basic remodeling features without evaluating possible mechanisms. We analyzed the effects of MSC therapy on lung vascular remodeling and hemodynamics and its possible mechanisms of action in monocrotaline (MCT)-induced PAH.

**Methods:**

Twenty-eight Wistar rats were randomly divided into two groups. In the PAH group, animals received MCT 60 mg/kg intraperitoneally, while a control group received saline (SAL) instead. On day 14, both groups were further randomized to receive 10^5^ adipose-derived MSCs or SAL intravenously (*n* = 7/group). On day 28, right ventricular systolic pressure (RVSP) and the gene expression of mediators associated with apoptosis, inflammation, fibrosis, Smad-1 levels, cell proliferation, and endothelial–mesenchymal transition were determined. In addition, lung histology (smooth muscle cell proliferation and plexiform-like injuries), CD68^+^ and CD163^+^ macrophages, and plasma levels of vascular endothelial growth factor (VEGF) and platelet-derived growth factor (PDGF) were evaluated.

**Results:**

In the PAH group, adipose-derived MSCs, compared to SAL, reduced mean RVSP (29 ± 1 vs 39 ± 2 mmHg, *p* < 0.001), lung tissue collagen fiber content, smooth muscle cell proliferation, CD68^+^ macrophages, interleukin-6 expression, and the antiapoptotic mediators Bcl-2 and survivin. Conversely, expression of the proapoptotic mediator procaspase-3 and plasma VEGF increased, with no changes in PDGF. In the pulmonary artery, MSCs dampened the endothelial–mesenchymal transition.

**Conclusion:**

In MCT-induced PAH, MSC therapy reduced lung vascular remodeling, thus improving hemodynamics. These beneficial effects were associated with increased levels of proapoptotic markers, mesenchymal-to-endothelial transition, reduced cell proliferation markers, and inflammation due to a shift away from the M1 phenotype.

**Electronic supplementary material:**

The online version of this article (doi:10.1186/s13287-017-0669-0) contains supplementary material, which is available to authorized users.

## Background

Pulmonary arterial hypertension (PAH) is a progressive disease, caused by a variety of pulmonary or cardiac disorders, which carries high rates of morbidity and mortality. PAH is characterized by a progressive increase in pulmonary arterial pressure, right heart dysfunction, and vascular remodeling, which leads to right ventricular failure [1–3]. Specific features of the vascular remodeling seen in PAH include apoptosis and proliferation of pulmonary vascular endothelial cells, muscularization of distal pulmonary arterioles, deposition of extracellular matrix proteins, and perivascular inflammation [2].

Several clinical [1, 4] and experimental [5–9] studies have sought to investigate the pathophysiology of PAH, which remains controversial [10]. Its characteristic perivascular inflammation involves interplay among several cell types, which include T and B lymphocytes, dendritic cells, mast cells, and macrophages. It is worth noting that perivascular macrophages—particularly the M1 phenotype—represent one of the main sources of IL-6 [11]. Although a heightened inflammatory state is clearly associated with PAH [2], the contribution of macrophage phenotypes to PAH pathophysiology remains unclear. Besides the role of inflammation, specific growth factors with proangiogenic effects may contribute to endothelial cell proliferation and fibroblast activation, which, in turn, contribute toward lung vascular remodeling [8, 10, 12].

The only currently available pharmacological therapies for PAH focus on vasodilatory effects, decreasing RV afterload, and relieving symptoms [13]. To date, no therapy has been able to minimize inflammation and vascular remodeling processes and thus modify the natural history of the disease.

Stem cell therapy may constitute a new treatment modality for PAH [14, 15]. Particularly, mesenchymal stromal cells (MSCs) may have the ability to secrete paracrine factors which can mitigate tissue damage [16]. Preclinical studies using MSCs in animals with PAH reported reduction in inflammation [16] and remodeling [17–20], which may be associated with improvement in hemodynamics [19]. However, those studies evaluated lung vascular remodeling based on either RV ratios [18, 19] or basic wall thickness [17, 20], instead of fulfilling histological criteria for PAH [21]. Furthermore, there is very little information about the cellular mechanisms (which includes macrophage subpopulations) and molecular factors [22] responsible for the beneficial effects of MSCs on right-heart hemodynamics in PAH.

The present study uses a rat model of monocrotaline-induced PAH to investigate the effects of MSC therapy on vascular histology and hemodynamics and to attempt to elucidate the possible mechanisms of MSC action in PAH, through analyses of macrophage subpopulations, expression of growth factors, levels of proapoptopic and antiapoptotic mediators, endothelial–mesenchymal transition, and cell proliferation.

## Methods

This study was approved by the Institutional Animal Care and Use Committee of the Health Sciences Centre, Federal University of Rio de Janeiro (CEUA 114/14), and registered with the Brazilian National Council for Animal Experimentation Control. All animals received humane care in compliance with the “Principles of Laboratory Animal Care” formulated by the National Society for Medical Research and the US National Academy of Sciences *Guide for the Care and Use of Laboratory Animals*. The present study followed the ARRIVE guidelines for reporting of animal research [23].

### Animal preparation and experimental protocol

On day 0, echocardiography analysis of the right ventricle was performed in 28 male Wistar rats (weight 235 ± 21 g), which were then randomized into two groups: monocrotaline-induced PAH (MCT, *n* = 14), in which animals received 60 mg/kg of MCT intraperitoneally (i.p.) (C2401; Sigma Chemical Co., St Louis, MO, USA); and control (CTRL, *n* = 14), in which animals received a similar volume of saline solution i.p. On day 14, cardiovascular function impairment was assessed by repeat echocardiography in the CTRL and MCT groups. Both groups were randomized again to receive either MSCs (*n* = 7, 10^5^ cells intravenously (i.v.) through the jugular vein diluted in 50 μl saline solution) or an equivalent volume of saline solution (SAL, *n* = 7). On day 28, echocardiography was repeated, hemodynamic parameters were measured, and animals were killed for postmortem analyses.

### Adipose cell culture

For isolation of adipose-derived MSCs, male Wistar rats were killed with sevoflurane (Sevorane® 2.0 vol.%; Cristália, Itapira, São Paulo, SP, Brazil) and the inferior vena cava was transected. Fat tissue around the epididymis was identified, removed, and placed in 1× phosphate-buffered saline (PBS). The tissue was then mechanically dissociated and placed in 2 ml of type 1 collagenase solution (1 mg/ml) for 35 minutes at 37 °C, with homogenization every 10 minutes. DMEM (Invitrogen Life Technologies, Grand Isle, NY, USA) supplemented with 10% fetal bovine serum (FBS; Hyclone, Rockford, IL, USA), 1% penicillin/streptomycin (Pen/Strep; Invitrogen Life Technologies), and 2 mM l-glutamine (Invitrogen) was added to the solution for collagenase inactivation, and the whole solution was centrifuged at 300 × *g* for 5 minutes. The pellet was transferred into tissue culture dishes containing the same medium. The culture medium was changed on the day after extraction and three times a week thereafter. MSCs were expanded in culture using Iscove’s Modification of Dulbecco’s Medium (IMDM; Hyclone), supplemented with 10% FBS, 10% horse serum (Hyclone), 1% Pen/Strep, and 2 mM l-glutamine; MSCs were used at the third to sixth passage. They were kept in culture at confluence no greater than 70%. Immediately before injections, cells were lifted using 2.5% Trypsin/EDTA (Invitrogen Life Technologies). Cell density and viability was determined using Trypan blue staining and counted using a hemocytometer. Cell pellets were then resuspended in 1× PBS to a final concentration of 1 × 10^5^ cells per 50 μl immediately prior to injection.

Approximately 1 × 10^5^ cells at the third passage were characterized as MSCs according to the International Society of Cellular Therapy Consensus; that is, adhesion to plastic under standard conditions, expression of specific surface markers (CD73, CD90, and CD105) and lack of others (CD11b, CD19, CD34, CD45), and ability to differentiate into mesenchymal lineages (such as adipocytes, chondrocytes, and osteoblasts) under in-vitro conditions [24]. For flow cytometry analysis, we used antibodies against CD90-PE (thymocytes), CD34-PE (hematopoietic precursors), and CD29-FITC (nonhematopoietic precursors) (BD Biosciences, USA). The absence of CD90 and CD34 and the presence of CD29 were used to identify MSCs [25]. The different MSC populations were further characterized by their capacity to differentiate into osteoblasts and chondroblasts. Osteogenic differentiation was induced by culturing MSCs for up to 3 weeks in DMEM 10% FBS and 15 mM HEPES (Sigma Chemical Co., St Louis, MO, USA), supplemented with 10^–8^ M/L dexamethasone (Sigma Chemical Co.), 5 μg/ml ascorbic acid 2-phosphate (Sigma Chemical Co.), and 10 mM/L β-glycerolphosphate (Sigma Chemical Co.). To observe calcium deposition, cultures were stained with Alizarin Red S (Nuclear, SP, Brazil). To induce chondrogenic differentiation, MSCs were cultured in DMEM supplemented with 10 ng/ml TGF-β1 (Sigma, St. Louis, MO, USA), 50 nM ascorbic acid 2-phosphate (Sigma Chemical Co.), and 6.25 mg/ml insulin for 3 weeks. To confirm differentiation, cells were fixed with 4% paraformaldehyde in PBS for 1 hour at room temperature, and then stained with Alcian Blue (pH 2.5) [26].

### Echocardiography

For echocardiographic assessment of cardiovascular function, rats were anesthetized with sevoflurane (Sevorane® 2.0 vol.%; Cristália), shaved over the precordial region, and examined with a Vevo 770 system (VisualSonics, Toronto, ON, Canada) coupled to a 30-MHz transducer. Images were obtained from parasternal views. Pulsed-wave Doppler was used to measure the pulmonary artery acceleration time (PAT) and pulmonary artery ejection time (PET), and the PAT/PET ratio was used as an indirect index of PAH. All parameters followed the American Society of Echocardiography and European Association of Cardiovascular Imaging guidelines, as validated in a previous article on noninvasive assessment of murine pulmonary arterial pressure [27, 28].

### Hemodynamic measurements

Rats were anesthetized with fentanyl 100 μg/kg i.p. (União Química, São Paulo, SP, Brazil) and midazolam 5 mg/kg i.p. (Dormicum, União Química, São Paulo, SP, Brazil). If required, additional anesthesia was provided by inhaled sevoflurane through a nasal device. Animals were placed in dorsal recumbency and the skin and muscle tissue of the anterior chest wall were removed until the ribs were visible. The thorax was then opened and a heparinized 19-G winged infusion set (Embramac, Itapira, SP, Brazil) was inserted into the RV. The RV systolic pressure (RVSP) and RV contraction rate (RV dp/dtmax) were assessed with a physiological data acquisition system (LabChart 7.0; ADInstruments, Sydney, Australia). Blood samples were collected through inferior vena cava puncture into a heparinized syringe. These samples were then centrifuged at 1500 × *g* for 5 minutes at 4 °C for plasma retrieval for enzyme-linked immunosorbent assay (see later). At the end of the experiment, animals were euthanized by i.v. injection of sodium thiopental 25 mg (Cristália) followed by complete exsanguination through transection of the abdominal aorta.

### Determination of pulmonary artery function

After hemodynamic measurements, the heart and lung were removed. The main pulmonary artery was carefully dissected and cleaned of connective tissue. Arteries were attached to a force transducer and placed in vertical chambers filled with modified Tyrode’s solution (123 mM NaCl, 4.7 mM KCl, 1.2 mM MgCl_2_, 1.2 mM KH_2_PO_4_, 15.5 mM NaHCO_3_, 1.2 mM CaCl_2_, and 11.5 mM dextrose), which were oxygenated and kept at 37 °C. After a stabilization period of 2 hours in 1.5 g tension, the solutions were exposed to increasing doses of phenylephrine (Phe, 1 nM–10 μM). After the contraction plateau, the arteries were exposed to increasing doses of acetylcholine (ACh, 1 nM–10 μM) to evaluate endothelial function by the maximum relaxation capacity.

### Quantification of lung collagen

The right upper lobe of the lung was homogenized in Tris–HCl 0.05 M and NaCl 1 M containing protease inhibitor (Hoffmann-La Roche) (pH 7.4). Total soluble collagen was extracted overnight at room temperature and quantified using the Sircol™ kit (Biocolor, Newton Abbey, UK). The results were expressed as micrograms of collagen/milligram of total protein by Bradford’s technique.

### Optical microscopy

For optical microscopy (OM) analysis, an additional 24 Wistar rats (*n* = 6/group) were subjected to the same protocol as already described. To keep lungs inflated before fixation, a similar end-expiratory pressure (3 cmH_2_O) was achieved among all groups by a brief period of mechanical ventilation (Flexivent; SCIREQ, Canada). Heparin (1000 IU) was injected into the tail vein to allow better visualization of lung structures under OM. To extract the lungs, a laparotomy was performed and the inferior vena cava transected to kill the animals by exsanguination. The left lung was then quickly isolated, frozen in liquid nitrogen, and placed in Carnoy’s solution. The lungs were dehydrated in a graded ethanol series and embedded in paraffin. Slices (4-μm thick) were cut, deparaffinized, and stained with hematoxylin–eosin (H&E). The histopathological features of the pre-acinar and intra-acinar pulmonary arteries were classified according to Pietra et al. [21] on the basis of: medial hypertrophy with increase in cross-sectional area; intimal thickening, defined as concentric laminar, eccentric, or concentric nonlaminar (both lesion types composed of fibroblasts and connective tissue matrix); and dilatation lesions. These histopathological features were graded on a five-point, semiquantitative, severity-based scoring system as follows: 0 = normal, 1 = changes in 1–25% of examined tissue, 2 = changes in 26–50% of examined tissue, 3 = changes in 51–75% of examined tissue, and 4 = changes in 76–100% of examined tissue. This semiquantitative five-point score has been validated in different animal models [26, 29]. Scoring was assessed independently by one coauthor (VLC), who is an expert in lung pathology and was blinded to group allocation.

Lung slices were also stained with the modified Russel–Movat method to highlight the muscular, connective, and elastic fibers of lung vessels in histological slides [30]. In brief, a mixture of five stains—Alcian Blue, Verhoeff hematoxylin, and Crocein Scarlet combined with acidic fuchsine and saffron—was used. At pH 2.5, Alcian Blue was fixed by electrostatic binding with the acidic mucopolysaccharides. Verhoeff hematoxylin has a high affinity for nuclei and elastin fibers, which are negatively charged. The combination of Crocein Scarlet with acidic fuchsine stains acidophilic tissue components in red. Then collagen and reticulin fibers were unstained by a reaction with phosphotungstic acid, and stained yellow by saffron. Smooth muscle hypertrophy and vascular endothelial growth factor (VEGF) expression were quantified using a weighted scoring system, as described elsewhere [31, 32]. Briefly, values from 0 to 4 were used to represent the severity of smooth muscle hypertrophy and VEGF expression, with 0 standing for no effect and 4 for maximum severity. Additionally, the extent of smooth muscle hypertrophy and VEGF expression per field of view was determined using values of 0–4, with 0 standing for no appearance and 4 for complete involvement. The final score for each feature was calculated as the product of extension and severity, ranging from 0 to 16. Scoring was assessed independently by one coauthor (VLC), who is an expert in lung pathology and was blinded to group allocation.

### Transmission electron microscopy of pulmonary arteries

Three slices measuring 2 mm × 2 mm × 2 mm were cut from three different segments of the right inferior lung. They were then fixed in 2.5% glutaraldehyde and phosphate buffer, 0.1 M (pH 7.4), for transmission electron microscopy (TEM) analysis (JEOL 1010 Transmission Electron Microscope; Japan Electron Optics Laboratory Co, Tokyo, Japan). For each TEM image (20 per animal), an injury score was determined. The following parameters were analyzed, considering the pulmonary arteries: medial hypertrophy and concentric laminar intimal thickening in the pre-acinar artery; and basement membrane thickening, myofibroblast proliferation, endothelial hyperplasia, and smooth muscle fibers in the intra-acinar artery. These parameters were graded on a five-point scoring system similar to that already described for histopathological features.

### Macrophage immunohistochemistry

Sections were deparaffinized and hydrated and the slides were incubated with 10 mM sodium citrate. Endogenous peroxidase activity was blocked with 3% hydrogen peroxide. Slides were washed in TBS with 0.05% Tween-20 (Sigma), blocked with serum-free protein block (Dako, Carpinteria, CA, USA), and immunostained with Vectastain ABC (Vector Laboratories, Inc., Burlingame, CA, USA). Antibodies against CD68 (1:100 dilution, 125212; Abcam), CD163 (1:100 dilution, 87099; Abcam), and NOS 2 (1:350 dilution, N-20; Santa Cruz Biotechnology) were incubated in TBS/Tween buffer overnight at 4 °C. Color was developed with 3,3′-diaminobenzidine tetrahydrochloride (Vector Laboratories, Inc.) and counterstained with H&E. An isotype immunoglobulin G (IgG) was used as negative control. CD68^+^ and CD163^+^ macrophage densities were assessed in five fields of view in the alveolar space by the point-counting technique, using a 100-point grid of known area (10^4^ μm^2^) attached to the eyepiece of the microscope [33]. The point-counting technique was assessed independently by one coauthor (VM), who was blinded to group allocation.

### Enzyme-linked immunosorbent assay

Plasma levels of VEGF and platelet-derived growth factor (PDGF) (PeproTech, Rocky Hill, NJ, USA) were quantified 14 days after MSC therapy or saline administration in the CTRL-SAL, CTRL-MSC, MCT-SAL, and MCT-MSC groups. The results were normalized by total protein content by Bradford’s technique, and expressed as picograms per microgram. All measurements were performed in accordance with manufacturer guidelines.

### Expressions of interleukin-6, interleukin-10, pro-caspase-3, Bcl-2, survivin, Smad-1, GSK3B, VE-cadherin, vimentin, and α-actin

Quantitative real-time reverse transcription polymerase chain reaction (RT-PCR) was performed to assess biological markers of inflammation (interleukin (IL)-6 and IL-10) and apoptosis (procaspase-3, Bcl-2, and survivin) in lung tissue, and levels of small mothers against decapentaplegic homolog 1 (Smad-1), glycogen synthase kinase-3 beta (GSK3β), and markers of endothelial–mesenchymal transition (VE-cadherin, vimentin, and α-actin) in pulmonary artery tissue. Central slices of the lungs and proximal sections of the pulmonary artery were cut, collected in cryotubes, flash-frozen in liquid nitrogen, and stored at –80 °C. Total RNA was extracted (RNeasy Plus Mini Kit; Qiagen, Hilden, Germany) and the RNA concentration measured by spectrophotometry (Nanodrop ND-1000 system; Thermo Scientific, Wilmington, DE, USA). First-strand cDNA was synthesized from total RNA (Quantitec Reverse Transcription Kit; Qiagen). Relative mRNA levels were measured with a SYBR green detection system in an ABI 7500 real-time PCR analyzer (Applied Biosystems, Foster City, CA, USA). Samples were run in triplicate. For each sample, the expression of each gene was normalized to the acidic ribosomal phosphoprotein P0 (*36B4*) housekeeping gene [34] and expressed as fold change relative to CTRL-SAL, using the 2^–ΔΔCt^ method [35, 36]:

ΔCt = Ct (reference gene) – Ct (target gene).

The following primers were used for lung tissue analyses: IL-6, 5′-CTC CGC AAG AGA CTT CCA G-3′ (forward) and 5′-CTC CTC TCC GGA CTT GTG A-3′ (reverse); IL-10, 5′-AGA AGG ACC AGC TGG ACA AC-3′ (forward) and 5′-GTC GCA GCT GTA TCC AGA GG-3′ (reverse); procaspase-3, 5′-GGC CGA CTT CCT GTA TGC-3′ (forward) and 5′-GCG CAA AGT GAC TGG ATG-3′ (reverse); Bcl-2, 5′-ATC GCT CTG TGG ATG ACT GA-3′ (forward) and 5′-TGA TTT GAC CAT TTG CCT GA-3′ (reverse); and survivin, 5′-AGC AGG TGG AAG AAC TGA CC-3′ (forward) and 5′-AAA GCA AAA CCC CAA ATC ATC-3′ (reverse). The following primers were used for pulmonary artery analyses: Smad-1, 5′-AGA ACC GAT TCT GCC TTG GG-3′ (forward) and 5′-ACT CCG CAT ACA CCT CTC CT-3′ (reverse); GSK3β, 5′-TCG CCA CTC GAG TAG AAG AAA′ (forward) and 5′-ACT TTG TGA CTC AGG AGA ACT-3′ (reverse); VE-cadherin, 5′-CAA TAC CGC CAA CAT CAC AG-3′ (forward) and 5′-TGG TGA GGA TGC ACA GAA AG-3′ (reverse); vimentin, 5′-CTG CCA AGA ACC TCC AGG AG-3′ (forward) and 5′-ACT TCG CAG GTG AGT GAC TG-3′ (reverse); and α-actin, 5′-GGA GAT GGC GTG ACT CAC AA-3′ (forward) and 5′-TTG CGT TCT GGA GGA GCA AT-3′ (reverse).

### Expression of TGF-β and type I and III procollagen in lung fibroblasts

Lung fibroblasts were extracted and RT-PCR was performed to measure biological markers associated with fibrosis (transforming growth factor (TGF)-β, type I procollagen (PC-1), and type III procollagen (PC-3)). Lung fibroblasts were isolated as described previously [37]. Briefly, the right upper lung was removed, cut into small fragments, digested enzymatically in Liberase solution at 37 °C for 30 minutes, rinsed three times with warm DMEM/F12 media with 10% FBS, 1× penicillin/streptomycin, centrifuged at 524 × *g*, resuspended in complete DMEM/F12 media, transferred to a tissue culture dish, and, finally, placed in a humidified tissue culture incubator at 37 °C and 5% CO_2_. After the cells reached 80–90% confluence, they were lysed and total RNA was extracted from frozen tissues using the RNeasy Plus Mini Kit (Qiagen), following the manufacturer’s recommendations. Again, the RNA concentration was measured by spectrophotometry in Nanodrop ND-1000 (Thermo Scientific, Wilmington, DE, USA). First-strand cDNA was synthesized from total RNA using a Quantitec reverse transcription kit (Qiagen). Relative mRNA levels were measured with a SYBR green detection system using ABI 7500 real-time PCR (Applied Biosystems, Foster City, CA, USA). Samples were measured in triplicate. For each sample, the expression of each gene was normalized to *36B4* housekeeping gene expression [34] and expressed as fold changes relative to CTRL-SAL, using the 2^–ΔΔ^Ct method described earlier.

The following primers were used: TGF-β, 5′-ATA CGC CTG AGT GGC TGT C-3′ (forward) and 5′-GCC CTG TAT TCC GTC TCC T-3′ (reverse); PC-1 (αI), 5′-AGA AGT CTC AAG ATG GTG GCC G-3′ (forward) and 5′-GGT CAC GAA CCA CGT TAG CAT C-3′ (reverse); PC-3 (αI), 5′-CAG CTA TGG CCC TCC TGA TCT T-3′ (forward) and 5′-GTA ATG TTC TGG GAG GCC CG-3′ (reverse); and 36B4, 5′-CAA CCC AGC TCT GGA GAA AC-3′ (forward) and 5′-CAA CCC AGC TCT GGA GAA AC-3′ (reverse).

### Statistical analysis

Each variable was tested for normality and variance using the Kolmogorov–Smirnov and Levene tests respectively. Data are presented as mean ± SD unless otherwise specified. On day 14, parametric data were compared by a one-sample Student’s *t* test (α < 0.05). For comparisons between MCT at 14 days vs MCT-SAL and MCT-MSC at 28 days, paired *t* tests were used (α < 0.05). On day 28, parametric and nonparametric data were analyzed by multiple Student *t* tests and Wilcoxon signed-rank tests respectively, followed by Bonferroni’s correction (adjusted α* < 0.0167 for three comparisons: CTRL-SAL vs CTRL-MSC, CTRL-SAL vs MCT-SAL, and MCT-SAL vs MCT-MSC). The Bonferroni-adjusted significance level was used to counteract multiple comparisons [38]. Pearson’s correlations were calculated and *p* < 0.05 was adopted as significant. All tests were performed in GraphPad Prism version 6.01 (Graph-Pad Software, La Jolla, CA, USA).

## Results

### Echocardiography and hemodynamic measurements

**Fig. 1:**
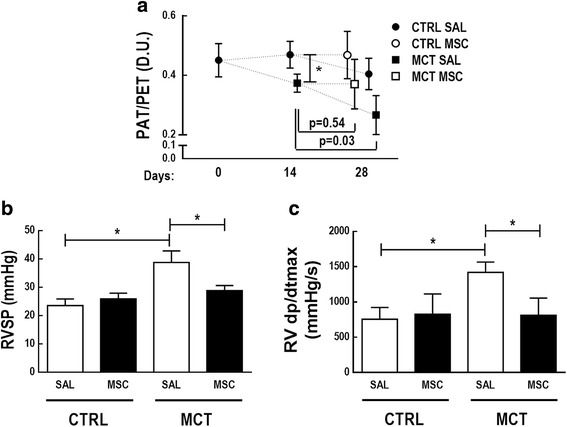
Progression of echocardiographic parameters. Echocardiography performed at 0, 14, and 28 days after injection of SAL (closed circle) or MCT (closed square). Open circle and square symbols represent CTRL and MCT animals respectively. (**a**) PAT/PET ratio. *p < 0.05. (**b**) Right ventricular systolic pressure (RVSP) at day 28. (**c**) Right ventricular contraction rate (RV dp/dtmax) at day 28. Values represent mean ± SD of seven animals/group. Comparisons by Student’s *t* test followed by Bonferroni’s procedure for three comparisons (*p < 0.0167). CTRL control, MCT monocrotaline, SAL saline, D.U. dimensionless units, PAT pulmonary acceleration time, PET pulmonary ejection time

Fourteen days after MCT induction of PAH, the PAT/PET ratio was reduced in PAH animals compared to CTRL, suggesting pulmonary hypertension (0.38 ± 0.09 vs 0.47 ± 0.08 respectively; *p* = 0.016). The ratio remained lower at day 28 (MCT-SAL, 0.27 ± 0.07 vs CTRL-SAL, 0.40 ± 0.05; *p* < 0.001). In the MCT-SAL group, the PAT/PET ratio was lower at day 28 compared to day 14 (*p* = 0.03). On the other hand, in the MCT-MSC group the PAT/PET ratio did not differ between days 14 and 28 (*p* = 0.54), which may suggest mitigation of the course of PAH (Fig. 1a).

On day 28, MCT-SAL animals exhibited higher RVSP (39 ± 2 mmHg vs 24 ± 1 mmHg respectively; *p* < 0.0001) (Fig. 1b) and RV dp/dtmax (1422 ± 144 mmHg/s vs 755 ± 166 mmHg/s respectively; *p* < 0.0001) (Fig. 1c) than CTRL-SAL animals. After MSC therapy, RVSP and RV dp/dtmax reduced in the MCT-MSC group compared to MCT-SAL (RVSP: MCT-MSC vs MCT-SAL, 29 ± 1 mmHg vs 39 ± 2 mmHg respectively, *p* < 0.001; RV dp/dtmax: MCT-MSC vs MCT-SAL, 812 ± 244 mmHg/s vs 1422 ± 144 mmHg/s respectively, *p* = 0.0006) (Fig. 1b, c).

### Endothelial dysfunction of pulmonary hypertension

Arteries from MCT-SAL animals exhibited less of a vasodilator response to increasing doses of ACh compared to those of CTRL-SAL animals (48 ± 5% vs 64 ± 2% respectively). This result was reinforced by calculation of the effective dose 50 (ED50) for ACh-induced dilation in both groups, in which CTRL-SAL animals showed a lower ED50 (–6.99) than MCT-SAL animals (–6.11). After MSC therapy, no additional vasodilator response of the pulmonary arterial rings was observed in MCT animals (Additional file 1: Figure S1).

### Evaluation of pulmonary arteriopathy

**Fig. 2:**
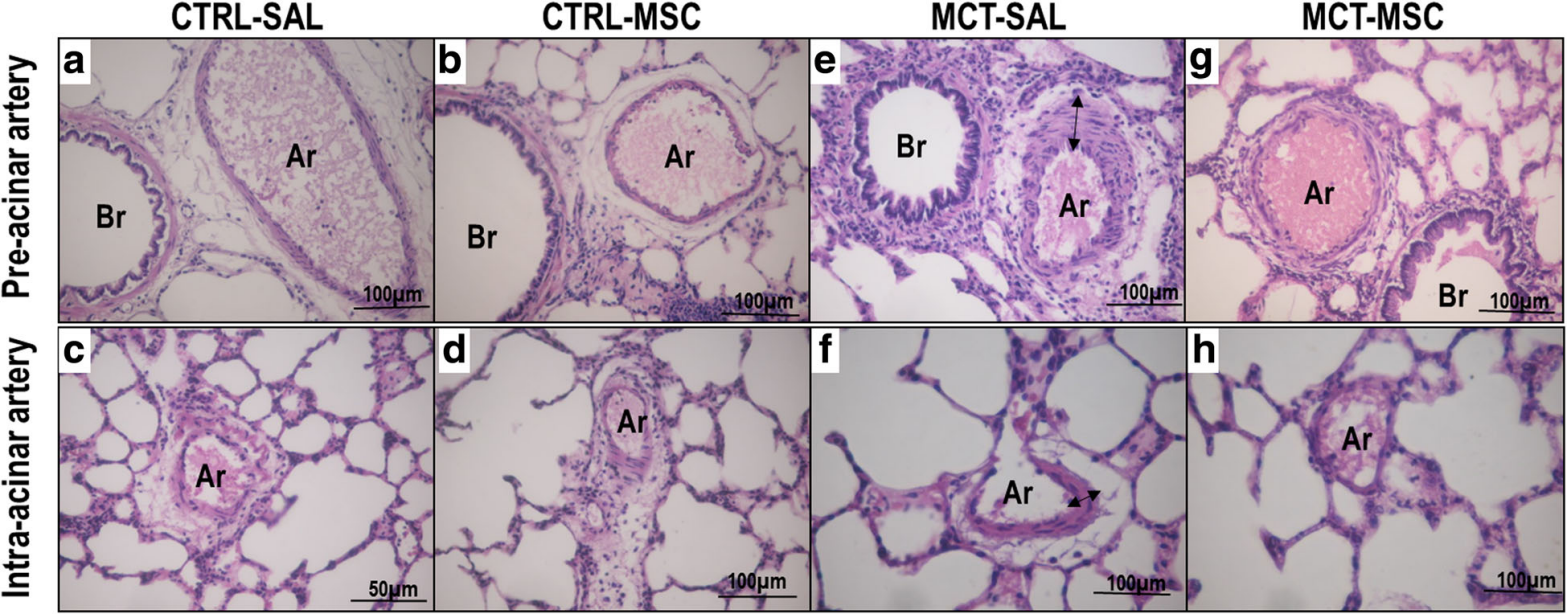
Main histopathological features of pulmonary arteriopathy. Normal medial thickening in CTRL-SAL and CTRL-MSC groups: (**a**, **b**) pre-acinar pulmonary artery; (**c**, **d**) intra-acinar artery. Concentric laminar intimal thickening in MCT-SAL animals: (**e**) pre-acinar artery (arrow); (**f**) intra-acinar artery (arrow). Decreased concentric laminar intimal thickening in MCT-MSC animals: (**g**) pre-acinar artery; (**h**) intra-acinar artery. CTRL control, MCT monocrotaline, SAL saline, Br bronchioles, Ar artery

On OM analysis, the following features were altered in MCT-SAL compared to CTRL-SAL animals: medial hypertrophy (pre-acinar, *p* = 0.006; intra-acinar, *p* = 0.01), concentric laminar intimal thickening (pre-acinar, *p* = 0.006; intra-acinar, *p* = 0.006), plexiform-like lesions (pre-acinar, *p* = 0.012; intra-acinar, *p* = 0.006), and dilatation lesions (pre-acinar, *p* = 0.006; intra-acinar, *p* = 0.006) (Table 1, Fig. 2). MSC therapy attenuated some histologic features, such as plexiform-like lesions in the pre-acinar artery (*p* = 0.008), concentric laminar intimal thickening in the intra-acinar artery (*p* = 0.008), and medial hypertrophy for both arteries (pre-acinar, *p* = 0.008; intra-acinar, *p* = 0.008) (Fig. 2).

**Table 1:** Histopathological score for pulmonary arterial hypertension

	Pre-acinar artery				Intra-acinar artery	
	Medial hypertrophy	Concentric laminar intimal thickening	Dilatation lesions	Medial hypertrophy	Concentric laminar intimal thickening	Dilatation lesions
CTRL-SAL	1 (1–1)	1 (1–1)	0 (0–0)	0 (0–0)	0 (0–0)	0 (0–0)
CTRL-MSC	1 (1–1)	1 (1–1)	0 (0–0)	0 (0–0)	0 (0–0)	0 (0–0)
MCT-SAL	4 (4–4)*	4 (4–4)*	2 (1–3)*	3 (2–4)*	3 (2–4)*	2 (2–2)*
MCT-MSC	2 (1–3)^†^	2 (2–2)	1 (0–1)	2 (1–3)^†^	2 (0–2)^†^	2 (1–3)

Classification of vasculopathies in pulmonary hypertension. Values presented as median (interquartile range) of seven animals/group

CTRL control, MCT monocrotaline, SAL saline

*Significantly different from CTRL-SAL group (*p* < 0.0167)

^†^Significantly different from MCT-SAL group (*p* < 0.0167)

On TEM analysis, MCT-SAL animals showed concentric laminar intimal thickening and prominent basement membrane in the pre-acinar and intra-acinar pulmonary arteries. In addition, MCT-SAL animals exhibited external fibronectin fibers and intracellular actin microfilaments. After MSC therapy, concentric laminar intimal and basement-membrane thickening decreased (Additional file 1: Figure S2).

**Fig. 3:**
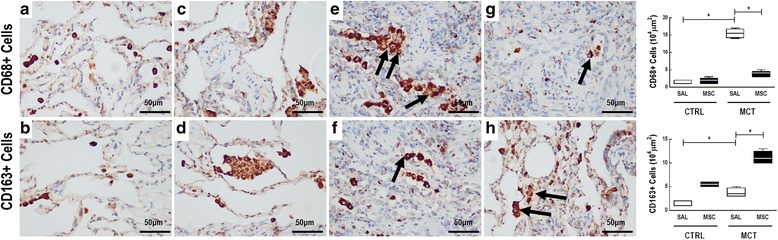
Representative immunohistochemistry photomicrographs and respective quantification of CD68^+^ and CD163^+^ macrophage density. Positive cell density determined as the number of positive cells by area unit (10^4^ μm^2^). **a**, **c** Very few CD68^+^ cells were observed. **e** CD68^+^ cells were observed (black arrows). **g** Few CD68^+^ cells were observed. **b**, **d**, **f**, Very few CD163^+^ cells were observed. **h** CD163^+^ cells were observed (black arrows). Lines represent medians, and whiskers are the interquartile range of seven animals in each group. Comparisons by Student’s *t* test followed by Bonferroni’s corrections for three comparisons (*p < 0.0167). CTRL control, MCT monocrotaline, SAL saline

MCT-SAL animals had higher CD68^+^ and CD163^+^ cell counts than CTRL-SAL (16 (14–17) vs 2 (1, 2), *p* < 0.0001; and 3 (3-4.75) vs 2 (1, 2), *p* < 0.0001 respectively). MCT-MSC animals, compared to MCT-SAL, exhibited reductions in CD68^+^ cell counts (4 (3–4.5) vs 16 (14–17), *p* = 0.016), as well as increased CD163^+^ cell counts (11 (10–12.5) vs 3 (3–4.75), *p* = 0.008) (Fig. 3).

**Fig. 4:**
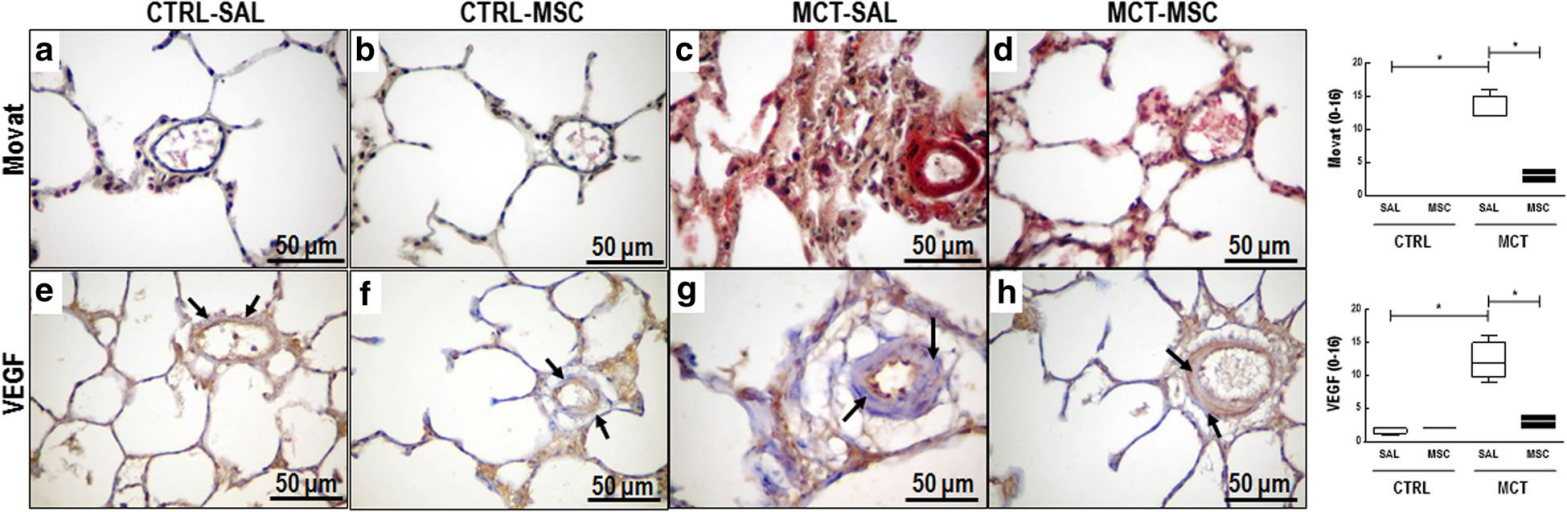
Main histopathological features of intra-acinar pulmonary arteriopathy induced by MCT. Histological sections stained with Movat (**a**–**d**). The vessel was decorated with anti-vascular endothelial growth factor antibody (VEGF) (**e**–**h**), revealing the endothelial expression (black arrow) to be composed of VEGF-positive cells. Intra-acinar artery: normal wall thickening and endothelial VEGF expression (arrows) in CTRL-SAL (**a**, **e**) and CTRL-MSC (**b**, **f**) respectively. Intra-acinar artery: hypertrophy and hyperplasia of smooth muscle fibers (**c**) and increased VEGF expression (**g**) in an MCT-SAL animal. Intra-acinar artery: attenuation of muscle cell hyperplasia (**d**) and VEGF expression (**h**) in an MCT-MSC animal. Lines represent medians, and whiskers are the interquartile range of seven animals in each group. Comparisons by Student’s *t* test followed by Bonferroni’s corrections for three comparisons (*p < 0.0167). CTRL control, MCT monocrotaline, SAL saline

VEGF content was higher in MCT-SAL animals than CTRL-SAL (12 (9.75–15) vs 2 (1.25–2) respectively; *p* = 0.002) (Fig. 4). Compared to SAL, MSC therapy was able to reduce the arteriolar VEGF content (*p* < 0.0001). MCT-SAL animals had higher smooth muscle cell hypertrophy and hyperplasia scores than CTRL-SAL animals (12 (12–15) vs 0 (0–0) respectively; *p* = 0.002). MSC therapy attenuated smooth muscle cell hypertrophy and hyperplasia, as demonstrated by significantly lower scores in MCT-MSC compared to MCT-SAL animals (*p* = 0.003) (Fig. 4).

### Quantification of lung collagen and mRNA expression of inflammatory, apoptotic, and profibrotic mediators and markers of cell proliferation and endothelial–mesenchymal transition

**Fig. 5:**
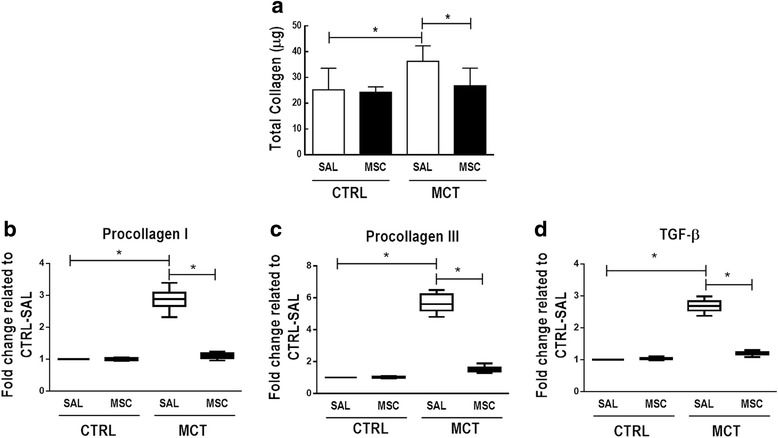
**a** Total amount of collagen in lung samples measured by colorimetric essay (Sircol™). Bars represent mean ± SD. Real-time polymerase chain reaction analysis for PC-1 (**b**), PC-3 (**c**), and transforming growth factor-β (TGF-β) (**d**) from lung fibroblast cell cultures. Lines represent medians, and whiskers are the interquartile range of seven animals in each group. Relative gene expression calculated as a ratio of the average gene expression level compared with the reference gene (*36B4*) and expressed as fold change relative to CTRL-SAL. Comparisons by Student’s *t* test followed by Bonferroni’s corrections for three comparisons (*p < 0.0167). CTRL control, MCT monocrotaline, SAL saline

MCT-SAL animals showed higher levels of collagen in lung tissue compared to CTRL-SAL animals (36 ± 2 μg vs 25 ± 3 μg respectively; *p* = 0.0145), and MSC therapy reduced collagen content in MCT animals (27 ± 3 μg vs 36 ± 2 μg respectively; *p* = 0.0169) (Fig. 5a).

MCT-SAL animals showed higher IL-6 (*p* < 0.0167) and survivin mRNA expression in lung tissue compared to CTRL-SAL. In addition, Smad-1 and GSK3β mRNA expressions were higher in MCT-SAL than CTRL-SAL animals (*p* < 0.0167 for both). In pulmonary artery tissue, VE-cadherin mRNA expression was lower, while vimentin and α-actin were higher in MCT-SAL animals compared to CTRL-SAL.

**Fig. 6:**
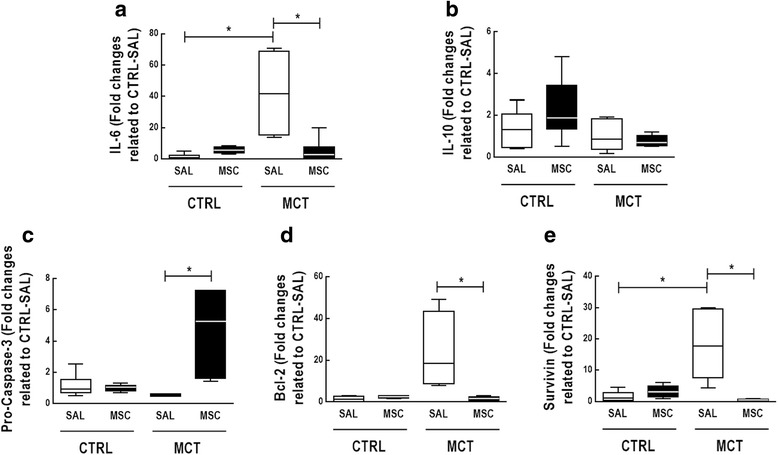
Real-time PCR analysis of biological markers associated with inflammation (interleukin (IL)-6, IL-10) (**a**, **b**), apoptosis (procaspase 3) (**c**), and antiapoptosis (B-cell lymphoma 2 (Bcl-2) and survivin) (**d**, **e**) in lung tissue. Lines represent medians, and whiskers are the interquartile range of seven animals in each group. Relative gene expression calculated as a ratio of the average gene expression level compared with the reference gene (*36B4*) and expressed as fold change relative to CTRL-SAL. Comparisons by Student’s *t* test followed by Bonferroni’s corrections for three comparisons (*p < 0.0167). CTRL control, MCT monocrotaline, SAL saline

**Fig. 7:**
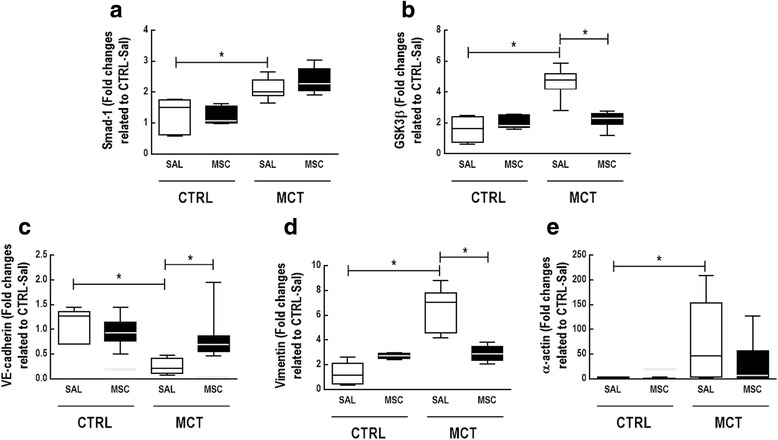
Real-time PCR analysis of small mothers against decapentaplegic homolog 1 (Smad)-1 (**a**), glycogen synthase kinase-3 beta (GSK3β) (**b**), VE-cadherin (**c**), vimentin (**d**), and α-actin (**e**) in pulmonary artery tissue. Lines represent medians, and whiskers are the interquartile range of seven animals in each group. Relative gene expression calculated as a ratio of the average gene expression level compared with the reference gene (*36B4*) and expressed as fold change relative to CTRL-SAL. Student’s *t* test followed by Bonferroni’s corrections for three comparisons (*p < 0.0167). CTRL control, MCT monocrotaline, SAL saline

MSC therapy decreased IL-6, Bcl-2, and survivin mRNA expression (*p* < 0.0167 for all) and increased procaspase-3 mRNA expression in lung tissue (*p* < 0.0167) (Fig. 6a–e). In pulmonary artery tissue, levels of GSK3β, but not Smad-1, mRNA were lower in MCT-MSC than in MCT-SAL animals (Fig. 7a, b). MCT-MSC animals showed increased VE-cadherin mRNA expression but reduced mRNA expression of mesenchymal markers such as vimentin and α-actin compared to MCT-SAL animals (Fig. 7c–e).

In lung fibroblasts, MCT-SAL compared to CTRL-SAL animals showed higher procollagen I, procollagen III, and TGF-β mRNA expressions, which were reduced in the MCT-MSC group in comparison to MCT-SAL (*p* < 0.0001) (Fig. 5b–d).

### Enzyme-linked immunosorbent assay for plasma VEGF and PDGF

On day 28, MCT-SAL animals had lower plasma VEGF levels compared to CTRL-SAL (29.5 ± 23.0 pg/μg vs 117.2 ± 39.4 pg/μg, *p* < 0.0167). In MCT-MSC animals, plasma VEGF was lower than in MCT-SAL (1.9 ± 1.3 pg/μg vs 29.5 ± 23.0 pg/μg, *p* < 0.0167). PDGF levels did not differ among groups (Table 2).

**Table 2:** Plasma levels of biomarkers

	CTRL		MCT	
	SAL	MSC	SAL	MSC
VEGF (pg/μg)	117.2 ± 39.4	135.9 ± 156.3	29.5 ± 23.0^#^	1.9 ± 1.3^†^
PDGF (pg/μg)	508.7 ± 205.2	520.7 ± 338.0	343.0 ± 239.0	458.1 ± 365.9

Plasma levels of vascular endothelial growth factor (VEGF) and platelet-derived growth factor (PDGF) at 28 days. Results normalized by total protein content (Bradford’s technique) as picograms per microgram. Values presented as mean ± SD of seven animals/group

CTRL control, MCT monocrotaline, SAL saline

^#^Significantly different from CTRL-SAL at 28 days (*p* < 0.0167)

^†^Significantly different from MCT-SAL at 28 days (*p* < 0.0167)

## Discussion

In the monocrotaline-induced experimental model of PAH used herein, MSC therapy reduced right ventricular systolic pressure, histological damage and smooth muscle cell thickening, CD68^+^ macrophage density in lung tissue, lung arteriolar and plasma VEGF levels, total lung collagen, mRNA levels of procollagen type I, type III, and TGF-β in lung fibroblast culture, and inflammatory and anti-apoptotic markers in lung tissue. MSC therapy increased proapoptotic mediators in lung tissue while decreasing cell proliferation markers in pulmonary artery tissue, suggesting minimization of the heightened proliferative state observed in monocrotaline-induced PAH. Furthermore, MSC therapy may act through the endothelial–mesenchymal transition, as measured by gene expression in the pulmonary artery.

Monocrotaline has been widely used to induce PAH in small rodents [6, 39, 40]. In this line, we observed several pathophysiological features associated with PAH in animals exposed to MCT, such as an increase in RV hypertrophy index (RV/LV + S; data not shown), RVSP, and medial hypertrophy and concentric laminar intimal thickening in both pre-acinar and intra-acinar arteries. Since we were concerned about the functional and histopathological features of PAH, the usage of MCT would fit with our research question, as well as with the respective time course alterations [41] after MSC treatment.

### Cell therapy and inflammation

In the present study, we used 10^5^ adipose-derived MSCs (a lower dosage than in previous studies [16]), obtained from healthy animals, and observed an attenuation of inflammatory markers, including IL-6, as in previous preclinical studies using MSCs in PAH animals. IL-6 is a key factor that facilitates a shift in pericytes from the quiescent state into their contractile, hyperproliferative state [42], which would increase pulmonary vascular resistance in PAH. Nevertheless, the association of IL-6 with macrophage polarization in PAH has not been clear. By modulating macrophage polarization and therefore IL-6 production, induced pluripotent stem cell (iPSC)-based therapy has been shown to reduce inflammation and M1 phenotype subpopulations in human polarized macrophages in vitro [11]. Herein, we observed not only IL-6 and CD68^+^ macrophage attenuation but also an increase in the CD163^+^ macrophage subpopulation in vivo. Although we did not evaluate the precise mechanism of mitigation of inflammatory process, it may rely on inhibition of the NF-κB pathway [11]. By modulating the M1 phenotype and IL-6 mRNA expression, MSCs may minimize the intense proliferation observed in PAH. In this line, we observed a reduction in mRNA expression of Bcl-2 and survivin, which are known to have antiapoptotic properties [43], leading to cell survival, and intense remodeling [44]. Likewise, we observed a relative increase in procaspase-3 mRNA expression after MSC therapy in MCT animals, which, by increasing apoptosis, may counteract the intense cell proliferation observed in PAH.

### Cell therapy and remodeling

Previous studies have reported a reduction in vascular remodeling after MSC therapy [16–18, 20]. Despite using a lower dose of MSCs than prior studies [16–20], we observed a reduction in the remodeling process. Furthermore, previous studies have focused only on pulmonary vessel wall thickness, which is a more general measurement [16–18, 20]. Conversely, we were interested in the main histopathological features of PAH seen in the pre-acinar and intra-acinar pulmonary arteries [21]. In the pre-acinar arteries, after MSC therapy, we observed a reduction in medial hypertrophy and plexiform-like lesions, which represent specific features in PAH [21, 45], while in the intra-acinar arteries there was a reduction in medial hypertrophy and concentric laminar intimal thickening. Similar results were observed after MSC therapy in atherosclerosis lesions [46]. In pulmonary artery smooth muscle cells, GSK3β represents a mediator of the Akt pathway toward cell proliferation [47]. It has been shown that GSK3β increases in monocrotaline-induced PAH, and its levels are negatively modulated by HIV protease inhibitors in line with decreased proliferation in pulmonary arteries [48]. We observed a reduction in GSK3β but not Smad-1 levels in pulmonary artery tissue after MSC therapy. This suggests that MSCs may interfere with the Akt–GSK3β axis, one of the major non-SMAD signaling pathways [49] toward cell proliferation [47]. Since Smad-1 belongs to the bone marrow protein receptor (BMPR)/Smad signaling pathway, the lack of effect observed after MSC therapy may be explained by the minor role of BMPR-2 in pulmonary artery smooth muscle cells in monocrotaline-induced PAH [50]. We also isolated fibroblast cells after MSC therapy in order to provide further support to cell therapy and remodeling. Fibroblast cells can change their phenotype into myofibroblasts, acting on mRNA expression of procollagen and TGF-β, which have been associated with intimal arterial injury during PAH [2] and intense endothelial-to-mesenchymal transition after MCT administration [51]. We observed a reduction in TGF-β, procollagen type I, and procollagen type III mRNA expression in lung fibroblast cell cultures after MSC therapy. Previous studies have demonstrated that MSC-conditioned medium significantly downregulates procollagen type I and procollagen type III mRNA expression, which reflects the paracrine antifibrotic effects of MSCs [52]. In addition, along with increased VE-cadherin, we also detected reductions in vimentin and α-actin, which suggests that the change from an endothelial into a mesenchymal phenotype observed in PAH [51] was dampened after MSC administration.

### Cell therapy and growth factors

Because of the controversial roles of different growth factors in PAH [53], we decided to evaluate VEGF and PDGF levels over time in plasma and small pulmonary vessels. VEGF is the most important factor regulating both physiological and pathogenic angiogenesis [54]. Serum and endothelial levels of VEGF, as well as VEGF receptor 2 (VEGFR-2) expression, are known to be increased in PAH patients [4, 55]. In PAH pathogenesis, VEGF may act as a potent stimulator of the disordered angiogenic process through a phenotype shift of pulmonary vascular smooth muscle cells from a quiescent state into a proliferative and antiapoptotic profile [7]. In this line, balancing this pathway might provide a desirable therapeutic avenue. In ischemia–reperfusion injury [56], MSC therapy has been shown to increase lung tissue VEGF levels, which was followed by vascularization and angiogenesis in vitro [56, 57]. In contrast, after MSC therapy, we observed lower VEGF levels in both plasma and small pulmonary vessels. The relationship between inflammation and angiogenesis may explain these contradictory findings. MSCs reduced inflammation by changing the macrophage phenotype from M1 (proinflammatory) toward M2 (anti-inflammatory) and, consequently, reducing IL-6 mRNA expression. In this scenario, lower proliferation and angiogenesis and, therefore, lower VEGF levels are to be expected. In addition, at the time of MSC administration (i.e., on day 14), circulating levels of VEGF were higher in MCT compared to CTRL animals. Administration of MSCs on day 14 would balance out the intense proliferative state and might act at the endothelial niche by the interaction of pericytes and the innate immune system [58], by increasing plasticity [59], and by balancing pulmonary vessel density [60].

In contrast, since no major alterations in plasma PDGF levels were observed, we did not evaluate PDGF in lung tissue after MSC therapy. Therefore, the observed reduction in proliferative status may be largely attributable to VEGF downregulation. It should be pointed out that the effects of MSCs may be attributable to their paracrine effects rather than to their presence in the injured lung area, since beneficial effects have been observed after administration of MSC-conditioned medium [61] and extracellular vesicles [62, 63] in PAH and other respiratory diseases.

### Cell therapy and functional parameters

In this experiment, MSC therapy may have improved hemodynamics, as represented by a reduction in RVSP, mainly due to attenuation of vascular remodeling [64]. Accordingly, we did not observe a significant vasodilatory response of the pulmonary arterial rings after MSC therapy, which provides further evidence of this effect. Previous studies have reported reductions in pulmonary arterial pressures after MSC administration, followed by a vasodilatory response to ACh [19]. The methods used in these prior investigations were based on the in-vivo setting, and involved i.v. injection of acetylcholine after administration of a prostaglandin agonist [19]. This has several limitations, including the fact that the systemic effects of ACh and prostaglandin might prevent proper measurement of endothelial dysfunction and that ACh dosages used in vivo are fourfold higher than those used in the present study during in-vitro measurements. In addition, the in-vitro method employed in our study has been widely used in prior research [65, 66].

### Limitations

The limitations of the present study must be taken into account. First, instead of performing repeated punctures for direct (invasive) measurement of RV pressures, we used echocardiography to choose the best timing for therapeutic interventions, according to indirect but reliable markers of pulmonary hypertension, such as the PAT/PET ratio [27, 67, 68]. Second, monocrotaline-induced PAH is not able to mimic the entire complex features observed in human PAH. Nevertheless, it is a well-established model of moderately severe PAH [69] and has been widely used both to elucidate its pathophysiological mechanisms [70, 71] and to propose new therapeutic interventions [65]. Third, we chose to analyze the roles of VEGF and PDGF specifically because these mediators are associated with angiogenesis [8, 72], as shown in prior studies with the MCT experimental model [71] and in human patients with PAH [2]. Nevertheless, we cannot rule out a role for other growth factors.

## Conclusion

In experimental monocrotaline-induced PAH, MSC therapy improved hemodynamics by mitigating lung vascular remodeling. These beneficial effects seem to be associated with an increase in proapoptotic markers, modulation of endothelial–mesenchymal transition, and decrease in cell proliferation markers and inflammation due to reduction in the M1 macrophage subpopulation.

## Additional file

Additional file 1: Determination of pulmonary artery function and Transmission electron microscopy of pulmonary arteries. (DOCX 3690 kb)

## Abbreviations

Ach: Acetylcholine
CTRL: Control
GSKβ: Glycogen synthase kinase-3beta
H&E: Hematoxylin–eosin
i.p.: Intraperitoneally
IL: Interleukin
iPSC: Induced pluripotent stem cell
MCT: Monocrotaline
MSC: Mesenchymal stromal cell
OM: Optical microscopy
PAH: Pulmonary arterial hypertension
PAT: Pulmonary artery acceleration time
PC-1: Type I procollagen
PC-3: Type III procollagen
PDGF: Platelet-derived growth factor
PET: Pulmonary artery ejection time
Phe: Phenylephrine
RT-PCR: Real-time reverse transcription polymerase chain reaction
RV dp/dtmax: RV contraction rate
RV/LV + S: RV hypertrophy index
RVSP: Right ventricular systolic pressure
SAL: Saline
Smad-1: Small mothers against decapentaplegic homolog 1
TEM: Transmission electron microscopy
TGF: Transforming growth factor
VEGF: Vascular endothelial growth factor
