# Supplementary material for: Mesenchymal stromal cell therapy reduces lung inflammation and vascular remodeling and improves hemodynamics in experimental pulmonary arterial hypertension
Source: Stem Cell Res Ther. 2017 Oct 3;8:220. doi: 10.1186/s13287-017-0669-0 (PMC5627397; doi:10.1186/s13287-017-0669-0)
Supplement: Additional file 1: — Determination of pulmonary artery function and Transmission electron microscopy of pulmonary arteries. (DOCX 3690 kb) [file 13287_2017_669_MOESM1_ESM.docx]

**Supplemental Digital Content**

**Mesenchymal stromal cell therapy reduces lung inflammation and vascular remodeling and improves hemodynamics in experimental pulmonary arterial hypertension**

Lucas De Mendonça^1,2^#, Nathane S. Felix^1,2^#, Natália G. Blanco^1,2^, Jaqueline S. Da Silva^3^, Tatiana P. Ferreira^5^, Soraia C. Abreu^1,2^, Fernanda F. Cruz^1,2^, Nazareth Rocha^1,4^, Patrícia M. Silva^5^, Vanessa Martins^1,6^, Vera L. Capelozzi^6^, Gizele Zapata-Sudo^3^, Patricia R.M. Rocco^1,2^, Pedro L. Silva^1,2^

^1^Laboratory of Pulmonary Investigation, Carlos Chagas Filho Biophysics Institute, Federal University of Rio de Janeiro, RJ, Brazil

^2^National Institute of Science and Technology for Regenerative Medicine, Rio de Janeiro, RJ, Brazil

^3^Laboratory of Cardiovascular Pharmacology, Federal University of Rio de Janeiro, RJ, Brazil

^4^Department of Physiology, Fluminense Federal University, Niterói, RJ, Brazil

^5^Laboratory of Inflammation, Oswaldo Cruz Institute – Oswaldo Cruz Foundation, Rio de Janeiro, RJ, Brazil

^6^Laboratory of Histomorphometry and Lung Genomics, University of São Paulo Faculty of Medicine, São Paulo, SP, Brazil.

Figure S1

**Figure S1**: Pulmonary arterial rings were exposed to increasing doses of phenylephrine and acetylcholine. ED50: median effective dose for phenylephrine and acetylcholine. Values represent mean ± standard deviation (SD) of 7 animals/group. *p<0.0167.

Figure S2.

**Figure S2:** Representative transmission electron microscopy of pulmonary artery. Normal medial thickening in CTRL-SAL and CTRL-MSC: (A, D) Pre-acinar pulmonary artery (×5,000); (B, C, E, F) Intra-acinar artery (×5,000). Concentric laminar intimal thickening and prominent basement membrane in MCT-SAL animals: (G) Pre-acinar artery (arrow) (×200); (H, I) Intra-acinar artery (×20,000 and ×40,000). Decreased concentric laminar intimal and basement membrane thickening in MCT-MSC group: (J) Intra-acinar artery (×5,000); (K, L) Intra-acinar artery (×20,000 and ×40,000). The fibronexus (L) comprises external fibronectin fibers (Fib) and intracellular actin microfilaments (Mac). The plasmalemma (arrows) within this fibronexus is visible at the tip of the myofibroblast (arrows). The fibronexus can be clearly identified in group MCT-SAL and is absent in the remaining groups. Ar: artery; End: endothelium; My: myofibroblast; BM: basement membrane. Arrows: concentric laminar intimal thickening. Stars: smooth muscle fibers.
